# QTL Analysis of Head Splitting Resistance in Cabbage (*Brassica oleracea* L. var. *capitata*) Using SSR and InDel Makers Based on Whole-Genome Re-Sequencing

**DOI:** 10.1371/journal.pone.0138073

**Published:** 2015-09-25

**Authors:** Yanbin Su, Yumei Liu, Zhansheng Li, Zhiyuan Fang, Limei Yang, Mu Zhuang, Yangyong Zhang

**Affiliations:** 1 Institute of Vegetables and Flowers, Chinese Academy of Agricultural Sciences, Beijing 100081, People’s Republic of China; 2 Beijing Zhongnong Futong Horticulture Corporation Limited, Beijing 100083, People’s Republic of China; Mahatma Phule Agricultural University, INDIA

## Abstract

Head splitting resistance (HSR) in cabbage is an important trait closely related to both quality and yield of head. However, the genetic control of this trait remains unclear. In this study, a doubled haploid (DH) population derived from an intra-cross between head splitting-susceptible inbred cabbage line 79–156 and resistant line 96–100 was obtained and used to analyze inheritance and detect quantitative trait loci (QTLs) for HSR using a mixed major gene/polygene inheritance analysis and QTL mapping. HSR can be attributed to additive-epistatic effects of three major gene pairs combined with those of polygenes. Negative and significant correlations were also detected between head Hsr and head vertical diameter (Hvd), head transverse diameter (Htd) and head weight (Hw). Using the DH population, a genetic map was constructed with simple sequence repeat (SSR) and insertion–deletion (InDel) markers, with a total length of 1065.9 cM and average interval length of 4.4 cM between adjacent markers. Nine QTLs for HSR were located on chromosomes C3, C4, C7, and C9 based on 2 years of phenotypic data using both multiple-QTL mapping and inclusive composite interval mapping. The identified QTLs collectively explained 39.4 to 59.1% of phenotypic variation. Three major QTLs (*Hsr 3*.*2*, *4*.*2*, *9*.*2*) showing a relatively larger effect were robustly detected in different years or with different mapping methods. The HSR trait was shown to have complex genetic mechanisms. Results from QTL mapping and classical genetic analysis were consistent. The QTLs obtained in this study should be useful for molecular marker-assisted selection in cabbage breeding and provide a foundation for further research on HSR genetic regulation.

## Introduction

Cabbage (*Brassica oleracea* L. var. *capitata*) is of immense importance for human nutrition, providing dietary fiber, vitamins, and cancer-preventing substances [1–2], and is one of the world’s most widely cultivated vegetables. During later stages of vegetative growth, cabbage heads are vulnerable to cracking. The appearance, yield, storability, and mechanical harvestability are seriously affected. In addition, susceptibility to head splitting hinders the prolongation of harvest time and thus the ability of growers to select harvest times for optimal selling price. Head splitting resistance (HSR) is thus a very desirable property in cabbage [3]. To alleviate head splitting, farmers generally reduce irrigation times during late-stage cultivation. Such practices affect normal cabbage head growth, however, and cannot completely prevent head splitting. Improvement of HSR in newly developed varieties has therefore become a priority in cabbage breeding programs.

HSR is believed to be complex and controlled by many genes. Gene action was found to be mostly additive and partial dominance for early splitting was detected [4–6]. Previous classical genetic methods can estimated the total gene effect, however, major gene effects and polygene effects were not clearly distinguished and the gene relationships were not identified. It is strongly recommended for plant breeders to apply major gene/polygene genetic segregation analysis as a simple and useful technique without any extra requirements on lab conditions except a precise experiment. Segregation analysis and quantitative trait locus (QTL) mapping are the main approaches used to clarify the genetic basis of quantitative traits [7–8]. These methods have been successfully applied to uncover inheritance patterns and QTLs of various plant systems [9–15].

Cabbage linkage maps with different types of markers have been reported previously. Before 2000, restriction fragment length polymorphism (RFLP), amplified fragment length polymorphism (AFLP), and random amplified polymorphic DNA (RAPD) markers were mainly used [16–20]; then from the beginning of 21st century, simple sequence repeat (SSR) makers were widely used because they were highly reproducible, highly polymorphic, and amenable to automation [21–23]; and during the last five years, single nucleotide polymorphism (SNP) [24–25], and insertion–deletions (InDels) gradually have become more favored as the genome sequence of cabbage was made available and the rapid development of next-generation sequencing. However, SNPs are costly and some special equipment is required for high-throughput genotyping [26]. As for InDel markers, they are obtained easily with the massive re-sequencing data through bioinformatic methods and also the electrophoresis bands are more credible with single distinguishable bands of about 100–200 bp, which reduces the genotyping errors greatly [27]. However, despite these obvious advantages, there were very little examples of application of InDel markers in cabbage breeding. QTL mapping studies in cabbage have mainly concentrated on disease resistance [28–30], agronomic trait [31], and flowering time [32]. QTL mapping for HSR in cabbage has only been reported by Pang et al. [33].

To thoroughly dissect the genetic architecture of HSR in cabbage, we first designed InDel markers resulting from the whole-genome re-sequencing data of the two parental lines. We then constructed a linkage map using these InDel and SSR makers designed based on the reference genome sequence with an intra-crop ‘immortal’ DH mapping population which was also used in a segregation analysis over multiple generations (P_1_, P_2_, and DHs) to explain HSR inheritance. Finally, we located the QTLs for HSR on the generated linkage map and analyzed QTL stability across years with different mapping programs. This information may increase the understanding on the genetic base and molecular mechanism of HSR trait and it is useful in order to identify genes controlling HSR in cabbage.

## Materials and Methods

### Plant materials and field experiments

**Fig 1:**
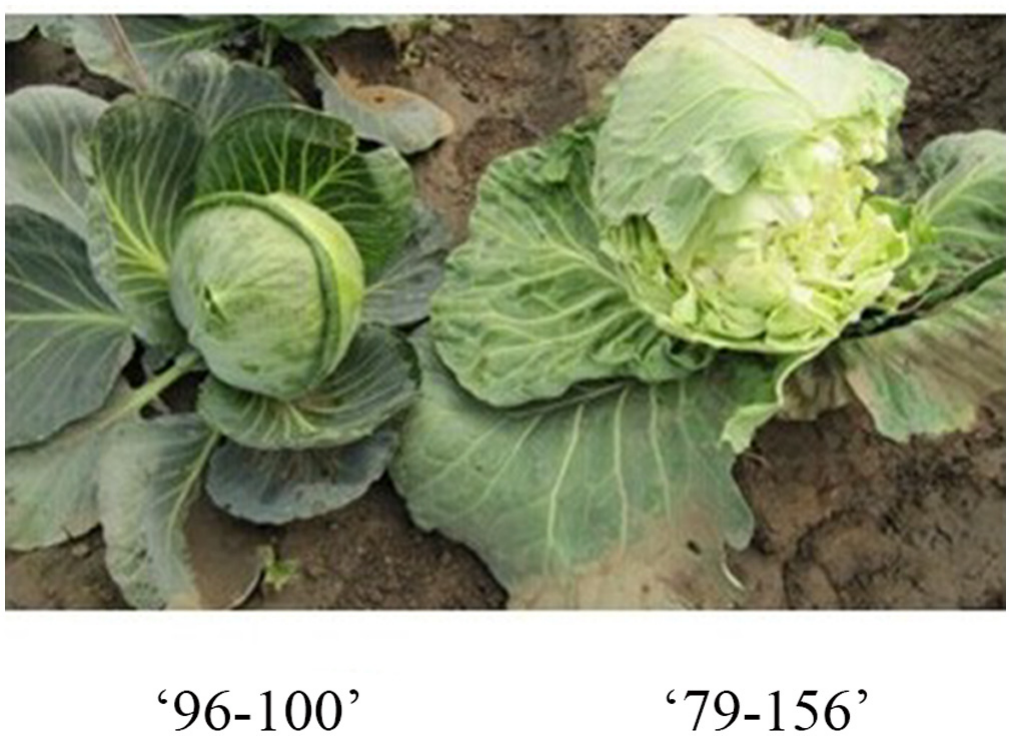
The difference between parents in head splitting resistance trait.

The female parental line, 79–156 (P_1_), is an inbred line self-developed from germplasm introduced from Denmark and is susceptible to head splitting; the male parental line, 96–100 (P_2_), is resistant to head splitting and was self-developed from germplasm of Indian origin introduced by the Bejo Sheetal Company (Fig 1). A double haploid (DH) population was derived from the F_1_ (79–156 × 96–100) generation by microspore culture [34–35] during 2009–2010. Ploidy levels of the regenerants were determined by flow cytometry or by detection of sterile and fertile plants as inferred by inflorescence, mature pollen grain production, and seed set. Haploid plants were subjected to *in vivo* diploidization with 0.2% colchicine solution for 10 h immediately prior to transplantation into soil in a greenhouse.

All generations were planted in the field at the experimental farm (Changping, Beijing, China) in autumn 2011 and 2012. Parental, F_1_, and RF_1_ (96–100 × 79–156) lines were planted in a randomized complete block design with three replicates and each replicate comprised 48 plants, spaced 40 cm apart within a row and 50 cm between rows. For the DH population, a block in replication design was adopted with three replicates [36] (S1 Table). Each replicate consisted of single rows of 16 plants of each DH line. The experimental plots were surrounded by two additional rows planted to serve as a protective buffer. The seedings were watered immediately after transplanted into the field, then they were watered at intervals of about 7 days. The irrigation frequency was reduced gradually at intervals of about 10 days since mid-October. In each replicate, 15 plants at the same growth level of each parental, F_1_, and RF_1_ line and 10 plants of each DH line were marked for phenotypic measurements.

### Resistance evaluation and statistical analysis

**Fig 2:**
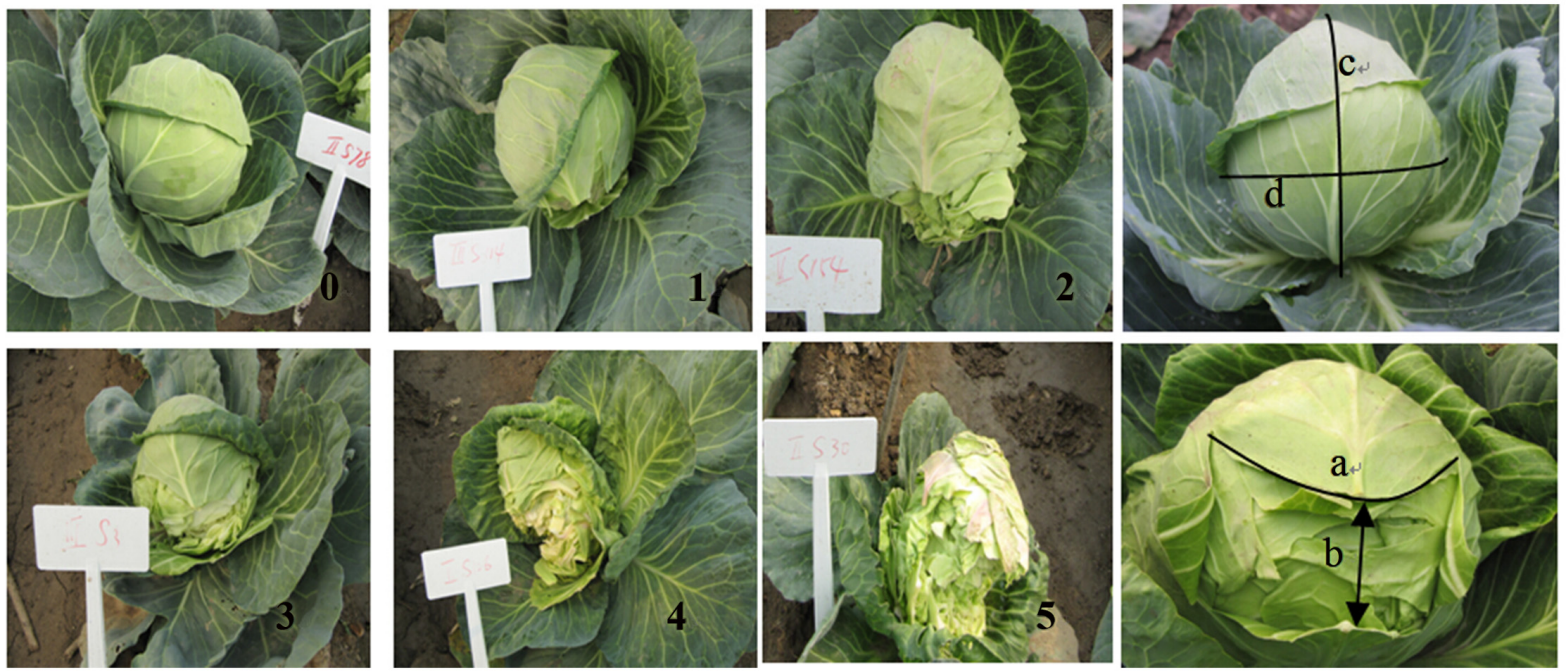
The grade 0–5 of head splitting resistance in DH population. **0** = no split; **1** = 1 split layer; **2** = 2 split layers and S < 50; **3** = 3–5 split layers and S < 50, or 2 split layers and S ≥ 50; **4** = 6–10 split layers and S < 50, or 3–5 split layers and S ≥ 50; **5** = more than 10 split layers, or 6–10 split layers and S ≥ 50. **a** = arc length and **b** = width of the largest split; **c** = height of the head and **d** = the largest circumference of the head. S [%] = S_1_/S_2_ × 100%, where S_1_ = a × b and S_2_ = c × 1/2 d.

Because of differences in maturation rates due to genotypic variation, maturation dates were recorded for each DH line. At maturity, the height and the circumference of each cabbage head at its widest point were measured with a tape measure. The arc length and width of the largest split were also measured with a tape measure 15 days after maturity. Splitting was assessed on a six-point scale, assigned according to the number of split layers and the ratio of the split size to the entire surface area (calculated as S [%] = S_1_/S_2_ × 100%, where S_1_ = arc length × width of the largest split and S_2_ = height × half of the largest circumference of the head) (Fig 2). Assigned splitting scores were as follows: 0 = no split; 1 = 1 split layer; 2 = 2 split layers and S < 50; 3 = 3–5 split layers and S < 50, or 2 split layers and S ≥ 50; 4 = 6–10 split layers and S < 50, or 3–5 split layers and S ≥ 50; 5 = more than 10 split layers, or 6–10 split layers and S ≥ 50. The head splitting index was calculated as: ∑ (splitting score × number of plants with that score) / (total number of plants × highest possible splitting score) × 100%. Each line was categorized with respect to HSR based on the head splitting index as follows: 0–5.0%, highly resistant; 5.1–15.0%, resistant; 15.1–35.0%, moderately resistant; 35.1–50.0%, susceptible; and >50.0%, highly susceptible. The other head trait evaluation was performed according to the following standards [37] :head mature period (Hm): days from transplanting to harvesting; head weight (Hw): weight of the matured cabbage head; head vertical diameter (Hvd): height from the base to the top of a matured cabbage head; head transverse diameter (Htd): length of the most breadth of a matured cabbage head; in addition, head shape index (Hsi: ratio of Hvd to Htd) was calculated. The data were analyzed with SAS 8.1 and SPSS 12.0 (SPSS Inc., Chicago, IL, USA) software.

### Joint segregation analysis

To determine an appropriate genetic model for the HSR trait, the cabbage DH population dataset was analyzed by major gene/polygene mixed inheritance analysis [38–39]. Joint segregation analysis was performed following Hao et al. (2008) and Zhang et al. (2010) under the basic assumptions described by those authors [10,14]. To select the genetic model best explaining the quantitative trait variation, 38 genetic models of 7 different types were considered (S2 Table). Maximum likelihood estimates of component parameters in each genetic model were generated using the iterated expectation and conditional maximization (IECM) algorithm [7]. The proportion, mean, and variance of each component distribution in the likelihood function were included in the estimates. Best-fit models were chosen according to the Akaike information criterion (AIC) [40] and a suite of goodness-of-fit tests. Finally, genetic parameters of major genes and polygenes were estimated based on the least squares principle [38–39] based on the distribution parameters of each component in the optimal model.

### DNA extraction and DNA mark analysis

Total DNA was isolated from expanding leaves of 3-week-old plants using the modified cetyltrimethylammonium bromide method [41]. The genomic DNA samples were diluted to 100 ng/μl with Tris-EDTA (pH 8.0) and stored at −20°C for use as polymerase chain reaction (PCR) templates. In addition, leaf tissue was lyophilized for use in future DNA extractions.

The cabbage 02–12 genome sequence of *B*. *oleracea* was retrieved from BRAD, the genomic database for *Brassica* (available at http://brassicadb.org), and used as the reference sequence. A total of 8.91 and 9.42 Gb Illumina pair-end reads were generated for the parental lines 79–156 (P_1_) and 96–100 (P_2_), respectively, using the sequencing-by-synthesis method. The reference sequence was used as a ‘bridge’ to detect sequence polymorphisms between the parental lines and 149 pairs of InDel primers were designed with the method by Liu et al. and Lv et al. [42–43]. In addition, a set of 2,170 SSR markers developed from cabbage sequence scaffolds [24] and 1,013 expressed sequence tag (EST)-SSR markers selected from the 62,567 *B*. *oleracea* ESTs in the National Center for Biotechnology Information database [44] were used to scan for polymorphisms between the two parents. DNA amplification and electrophoresis were performed according to Lv et al. [43].

### Linkage map construction and QTL analysis

For map construction, the DH population was genotyped for all SSR and InDel markers that showed polymorphisms between the parental 79–156 and 96–100 lines. The genotyping data were coded as type ‘a’ or ‘b’, corresponding to parental lines 79–156 and 96–100, respectively, with ambiguous and missing data indicated by ‘-’. A linkage map was constructed from the DH genotyping data using JoinMap 4.0 software [45]. For map distance calculations, recombination frequencies were converted to centiMorgans (cM) using Kosambi’s method, and linkage groups were assigned to chromosomes C1–C9 of *B*. *oleracea* based on markers in common with the reference [24, 43].

QTLs were estimated by multiple-QTL modeling (MQM) with MapQTL 4.0 [46] and by inclusive composite interval mapping (ICIM) using QTL IciMapping v3.0 software [47]. In MQM, permutation tests were first conducted on this DH population for 1000 times. LOD threshold was 3.28 under type I error 0.05. Interval mapping at 1-cM intervals along the chromosomes was then used to scan for QTLs based on a logarithm of odds (LOD) threshold of 3.28. Markers closely linked to positions with the highest LOD score were taken as cofactors for MQM analysis. To select significant markers during the first step of ICIM stepwise regression, *P*-values for entering and removing variables were set respectively at 0.001 and 0.002; in the second step, a minimum LOD threshold of 3.28 was used to declare a QTL significant.

## Results

### Production of the DH mapping population

Microspore culture of short (2.8–3.2 mm) buds from the F_1_ (79–156 × 96–100) generation yielded 1,052 normal embryos, with 4–10 embryos generated per bud. After plant regeneration and identification of ploidy levels, we obtained 584 plants consisting of 226 haploids, 294 doubled diploids, 10 polyploids, and 54 chimeras. The spontaneous recovery rate of DH individuals was 50.3%, which is typical for *B*. *oleracea* [48]. Not all individuals survived the culture process; of those that did, not all successfully produced seed. Seeds were obtained from 157 DH individuals in the summer of 2011 and from 181 individuals in 2012. All DH lines obtained in the 2 years were used for genetic segregation analysis. For linkage map construction and mapping, data from the first 157 lines were used.

### Phenotypic assessment of parental, F1, RF1, and DH lines

Parents 79–156 and 96–100 differed significantly with respect to HSR (Fig 1; Table 1). Line 96–100 was resistant to head splitting, with a head splitting index of 9.33 and 9.36 in 2011 and 2012, respectively; in contrast, 79–156 was highly susceptible to head splitting, with corresponding head splitting index values of 86.87 and 85.32. Head splitting indexes of F_1_ and RF_1_ were not significantly different from one another; the values were intermediate to those of the parents, indicating that no cytoplasmic effect on HSR inheritance was present in cabbage.

**Table 1:** Statistical summary of head splitting resistance in parents, F_1_, RF_1_, and doubled haploid (DH) populations.

Head-splitting index	Parents, F_1_ and RF_1_				DH populations				
	79–156	96–100	F_1_	RF_1_	Mean	SD	Variation range	Skewness	Kurtosis
2011	86.87c*	9.33a	30.50b	31.10b	35.16	28.27	0.00–98.52	0.76	-0.73
2012	85.32c	9.36a	33.12b	32.28b	30.45	28.55	0.00–100	0.97	-0.29

* Values within a given row followed by the same lowercase letter are not significantly different (*P* < 0.05) according to Duncan’s multiple range test.

Analysis of variance (ANOVA) revealed significant differences (*P* < 0.01) in HSR among DHs in both 2011 and 2012 (Table 2), indicating the existence of heritable variation and thus the suitability of the DHs for genetic analysis. Significant differences between years and among lines were observed at *P* < 0.05 and *P* < 0.01 levels, respectively; this result demonstrates that the HSR trait is mainly under genetic control, with climate also playing an important role.

**Table 2:** Analysis of variance of HSR in the doubled haploid population.

Year	Source	*DF*	*SS*	*MS*	*F*
2011	block^a^	13	44468.71	3420.67	152.81
	line	141	346887.36	2460.19	109.91**
	replication	2	89.02	44.51	1.99
	block× rep	26	557.36	21.44	0.96
	error	282	6312.42	22.38	
	corrected total	464	398314.87		
2012	block	10	41866.24	4186.62	117.53
	line	169	402077.30	2379.16	66.79**
	replication	2	95.09	47.54	1.33
	block × rep	20	664.32	33.22	0.93
	error	338	12040.46	35.62	
	corrected total	539	456743.42		
2011 and 2012	year	1	784.70	784.70	5.78*
	line	154	236825.86	1537.83	11.34**
	error	154	20891.50	135.66	
	corrected total	309	258502.06		

* Significant differences at *P* < 0.05.

** Significant differences at *P* < 0.01.

^a^ Doubled haploid (DH) lines were first divided randomly into 14 blocks in 2011 and 11 blocks in 2012; the blocks were then distributed according to a randomized complete block design with three replicates.

**Fig 3:**
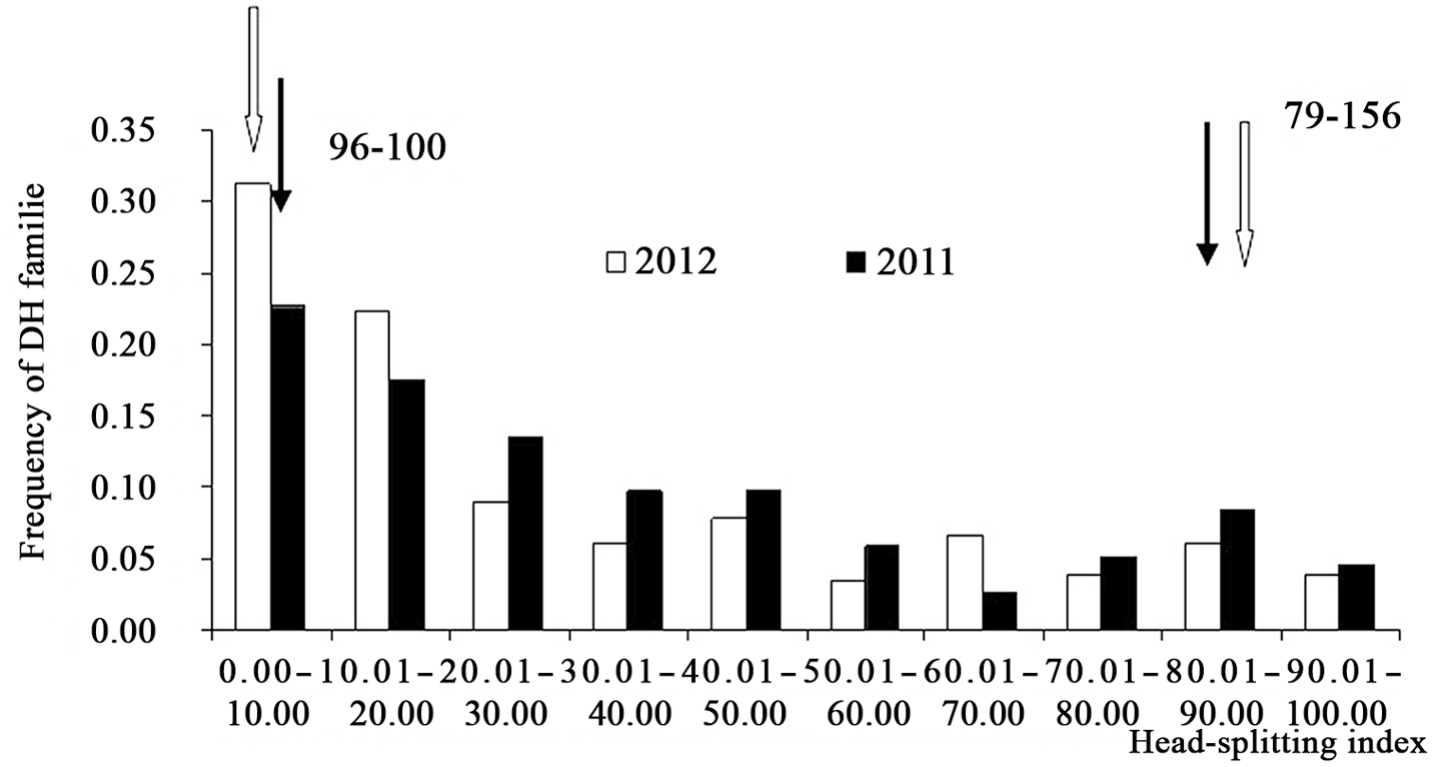
Frequency distribution of head-splitting index in DH families in 2011 and 2012. Arrows indicate the mean head-splitting index of the parental lines.

In the DH population, the head splitting index showed continuous variation, suggesting that comprehensive HSR in cabbage is a typical quantitative genetic character (Fig 3). Nevertheless, the frequency distribution of phenotypes deviated from a normal distribution, with skewness and kurtosis values of 0.76 and −0.73 in 2011 and 0.97 and −0.29 in 2012, respectively. These skewed and multi-peak phenomena indicate the possible existence of major genes for HSR. The head splitting index of DHs ranged from 0.00 to 98.52 in 2011 and 0.00 to 100.00 in 2012, with maximum values of the index much greater than in the parents. The observed transgressive segregation indicates that genes controlling HSR and having plus or minus effects are scattered throughout the genome and can produce extreme phenotypes in both positive and negative directions through gene recombination. In 2011 and 2012, the corresponding average head splitting index values were 35.16 and 30.46, with the distribution of the data in the DH population skewed toward the resistant parental type.

Pearson correlation tests were conducted for the six head traits to evaluate the correlations between Hsr and the other five traits (Table 3). Negtive and significant correlations were detected between Hsr and Hvd, Htd, or Hw. The highest correlation was -0.388 between Hsr and Hw, however, there was no correlation between Hsr and Hm/Hsi.

**Table 3:** Correlation (Pearson) analysis of 6 main head traits in DH population.

Traits	Hm	Hvd	Htd	Hsi	Hw	Hsr
Hm (day)	1	0.126	0.036	-0.282**	-0.379**	0.148
Hvd (cm)		1	0.790**	0.383**	0.852**	-0.308**
Htd (cm)			1	-0.258**	0.909**	-0.250**
Hsi				1	-.025	-0.104
Hw (kg)					1	-0.388**
Hsr						1

Hm head mature period, Hw head weight, Hvd head vertical diameter, Htd head transverse diameter, Hsi head shape index.

*Correlation is significant at the 0.05 level (2-tailed).

**Correlation is significant at the 0.01 level (2-tailed).

### Assessment of genetic models and genetic effect analysis

AIC scores of 38 specific models were obtained following IECM estimation and three possible models were selected according to the lower AIC criterion (S3 Table). The G-0 model, with the least number of significant parameters (0), was selected as the optimal genetic model for HSR analysis after a set of quantitative tests, including the *χ* ^2^ uniformity test (*U*_1_^2^, *U*_2_^2^, and *U*_3_^2^), Smirnov’s test (n*W* ^2^), and Kolmogorov’s test (*D*_n_) in both years (S4 Table). We therefore deduced that HSR in cabbage can be described by G-0 model corresponding to three pairs of additive-epistatic major genes plus additive-epistatic polygenes.

Maximum likelihood frequency values of eight component distributions were estimated under the G-0 model, with first- and second-order genetic parameters then calculated from the results (Table 4) using the least squares method. The calculated parameters showed similar tendencies in both years: additive effects (*d*) of the three major genes were estimated as 20.4, 8.59, and 15.89. All the additive effects were positive, indicating that the susceptible parental line 79–156 had important effects on total variability. The additive epistatic effect of the first two major genes, *i*_ab_, was 3.64, while that between the first and third major genes, *i*_ac_, was 10.94. The additive epistatic effect of the second and third major genes, *i*_bc_, was only −0.86, while that among the three major genes, *i*_abc_, was −5.81. Similar results were observed in 2012. Heritabilities of major genes and polygenes in 2011 were 88.03 and 7.60%, respectively, with corresponding values of 88.22 and 5.65% in 2012. The heritability values of the major genes were much larger than those of the polygenes. These observations demonstrate that HSR is mainly controlled by major genes, and that selection for this trait should be carried out in early generations.

**Table 4:** Estimates of genetic parameters under the G-0 model over 2 years.

Distribution parameter	Estimate		1^st^ order parameter	Estimate		2^nd^ order parameter	Estimate	
	2011	2012		2011	2012		2011	2012
*μ*_1_	90.57	90.62	*m*	37.78	32.93	*σ*_p_^2^	512.54	581.13
*μ*_2_	79.43	65.17	*d*_a_	20.4	20.13	*σ*_mg_^2^	451.22	512.70
*μ*_3_	32.23	12.80	*d*_b_	8.59	7.04	*σ*_pg_^2^	38.94	32.81
*μ*_4_	12.44	12.80	*d*_c_	15.89	12.42	*σ* ^2^	22.38	35.62
*μ*_5_	50.25	43.65	*i*_ab_	3.64	7.04	*h*_mg_^2^ (%)	88.03	88.22
*μ*_6_	12.44	12.80	*i*_ac_	10.94	12.42	*h*_pg_^2^ (%)	7.60	5.65
*μ*_7_	12.42	12.80	*i*_bc_	-0.86	-0.67			
*μ*_8_	12.42	12.80	*i*_abc_	-5.81	-0.68			

*μ*_1—_ *μ*_8_, eight component distributions; *m*, population mean; *d*_a_, *d*_b_, and *d*_c_, additive effects of the first, second, and third major genes, respectively; *i*_*ab*_, *i*_ac_, *i*_bc_, and *i*_abc_, interaction effect of the first and second major genes, the first and third major genes, the second and third major genes, and the three major genes, respectively; *σ*_p_^2^, phenotypic variation; *σ*_mg_^2^, major gene variation; *σ*_pg_^2^, polygenic variation; *σ* ^2^, environmental variation; *h*_mg_^2^, major gene heritability; *h*_pg_^2^, polygene heritability.

### Linkage map construction and analysis

A preliminary screening of 79–156 and 96–100 parental genotypes using a total of 3183 pairs of SSR and 149 pairs of InDel markers developed from cabbage sequence scaffolds, *B*. *oleracea* ESTs and the whole-genome re-sequencing data identified 372 polymorphic markers between the two parents. Of these polymorphic markers, in total 140 (4%) pairs of SSR primers and 101 (68%) pairs of InDel primers with reliable PCR products were selected to genotype the mapping DH population after removing ambiguous markers. The data was then analyzed using the JoinMap 4.0 software with a LOD threshold of 4.0 to construct the linkage map. The linkage analysis uncovered nine linkage groups which were designated as C1–C9 in accordance with the nomenclature used by Wang et al. (2012) [24] and Lv et al. (2014) [38] based on the presence of reference SSR markers and the physical positions of the InDel markers used in this study.

**Fig 4:**
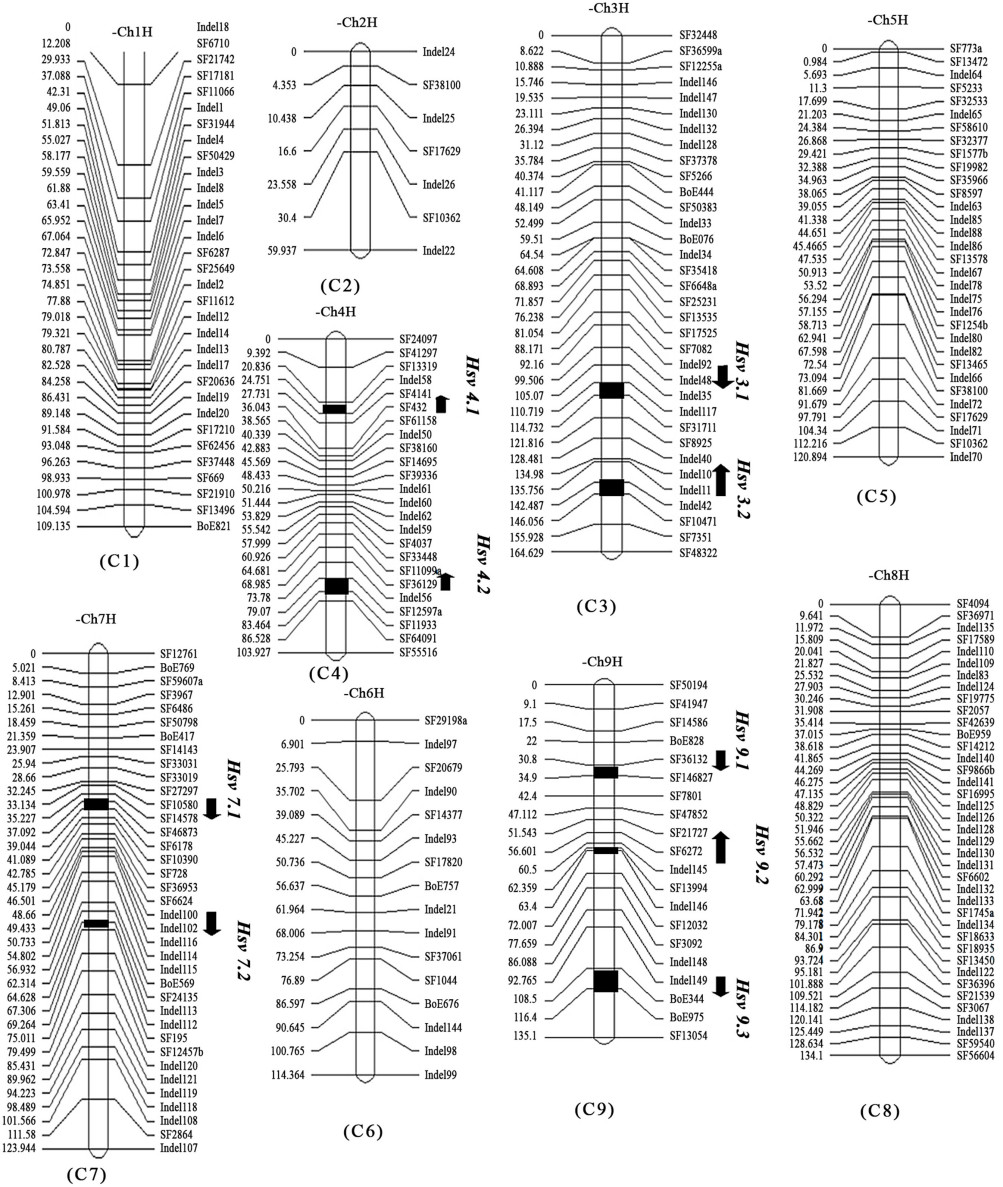
Genetic linkage map and positions of QTLs associated with heading splitting resistance in a cabbage DH population. Marker locations are listed to the right and recombination distances (cM) to the left of each linkage group. Locations of QTLs are indicated by names and arrows to the right of the linkage groups. Arrows indicate the relative effect of the 96–100 allele with upward for increasing and downward for decreasing.

The framework linkage map (Fig 4) was 1065.9 cM in length, with an average between-marker distance of 4.4 cM, a minimum between-marker distance of 0.55 cM, and a maximum distance of 29.5 cM. The largest linkage group, encompassing 164.6 cM, was C3; the smallest, C2, spanned 59.9 cM. The maximum average distance between markers (8.6 cM) was that of C2, which featured the lowest number of markers (3); the minimum average distance (3.3 cM) was found on C7. C8 had the highest number of markers (39). The cabbage genome has been variously estimated to comprise 603 [49] or 630 Mbp [50]. Using the latest estimate of Liu et al. (2014) [45], the average physical distance between mapped markers was calculated to be 2.61 Mbp.

Based on a χ^2^ test for goodness of fit to the expected 1:1 Mendelian segregation ratio, 140 of 241 loci (58%) displayed varying degrees of distortion (*P* ≤ 0.01). Although slightly more 79–156 alleles (60%) were present among the 140 distorted loci compared with 96–100 (40%) alleles, there was not a significant departure from a 1:1 Mendelian ratio. Linkage groups C1, C4, and C9 had clusters of markers that were distorted towards 96–100 alleles, whereas linkage groups C3, C5, C6, and C7 contained clusters of markers distorted in favor of 79–156. Linkage group C8 included small clusters of both parental genotypes. No heterozygous loci were scored during genotyping of the molecular markers.

### QTL mapping for HSR in cabbage

As shown in Table 5 and Fig 4, nine QTLs for HSR during 2 years were detected using MQM and ICIM methods with Map QTL 4.0 and IciMapping 3.0 software. The QTLs were located on cabbage chromosomes C3, C4, C7, and C9 and individually explained 5.50–16.93% of observed phenotypic variation. Using the MQM program, six QTLs were detected on chromosomes C4, C7, and C9; these QTLs collectively accounted for 41.6% and 39.4% of the phenotypic variation in 2011 and 2012, respectively, with the effect of each QTL ranging from 7.7 to 13.6% over the 2 years. *Hsr 4*.*2* and *Hsr 9*.*2*, which were robust QTLs showing a relatively large effect, could be detected in both years and were distributed between markers SF12597a/SF11933 and SF13994/Indel146. In both years, the 96–100 allele at the *Hsr 4*.*2* and *Hsr 9*.*2* locus increased the tendency toward HSR. Using ICIM, seven QTLs were detected on chromosomes C3, C4, and C9 and together explained 59.11% and 56.15% of observed phenotypic variation in 2011 and 2012, respectively. Their individual effects ranged from 5.5% to 16.93%—the same trend observed using MQM. *Hsr 3*.*2*, which were robust QTLs showing a larger effect, could be detected in both years and were distributed between markers Indel142/SF10471. Four of the QTLs (*Hsr 4*.*1*, *Hsr 4*.*2*, *Hsr 9*.*2*, and *Hsr 9*.*3*) either overlapped or were adjacent to the corresponding regions detected using MQM. *Hsr 4*.*2* and *Hsr 9*.*2* were also robust QTLs showing a relatively large effect, could be detected in both years. The locus *Hsr 3*.*2*, detected only by ICIM, was a major QTL explaining 15.36–16.93% of the phenotypic variation in both years. The 96–100 allele increased HSR at the *Hsr 3*.*2* locus in both years. The other QTLs which were detected only in one year or one method were minor genes.

**Table 5:** Analysis of quantitative trait loci (QTLs) for head splitting resistance in a cabbage doubled haploid population using multiple-QTL mapping (MQM) and inclusive composite interval mapping (ICIM) approaches.

Year	QTL names^a^	Linkage group	Position (cM)	LOD	Marker interval	*R* ^2^ (%)^b^	Add^c^
2011 (MQM)	*Hsr 4*.*2*	C4	80.0	5.46	SF12597a-SF11933	11.8	9.46
	*Hsr 7*.*1*	C7	37.2	3.42	SF46873-SF6178	7.7	-6.78
	*Hsr 7*.*2*	C7	68.0	3.74	Indel112-Indel113	8.5	-7.84
	*Hsr 9*.*2*	C9	63.2	5.62	SF13994-Indel146	13.6	10.94
2012 (MQM)	*Hsr 4*.*1*	C4	23.0	3.86	SF13319-Indel58	8.6	8.25
	*Hsr4*.*2*	C4	81.4	5.61	SF12597a-SF11933	12.8	10.78
	*Hsr 9*.*2*	C7	63.2	5.48	SF13994-Indel146	11.1	-10.02
	*Hsr 9*.*3*	C9	112.8	3.66	BoE344-BoE975	7.44	-6.92
2011 (ICIM)	*Hsr 3*.*1*	C3	112.0	3.36	Indel117-SF31711	5.50	-6.78
	*Hsr 3*.*2*	C3	143.0	7.31	Indel42-SF10471	16.93	11.76
	*Hsr 4*.*1*	C4	23.0	6.16	SF13319-Indel58	12.56	10.18
	*Hsr 4*.*2*	C4	80.0	6.96	SF12597a-SF11933	12.86	10.37
	*Hsr 9*.*2*	C9	63.0	5.74	SF13994-Indel146	11.26	9.76
2012 (ICIM)	*Hsr3*.*2*	C3	143.0	7.86	Indel42-SF10471	15.36	11.76
	*Hsr4*.*2*	C4	80.0	6.93	SF12597a-SF11933	11.1	9.15
	*Hsr 9*.*1*	C9	34.0	4.62	SF36132-SF146827	9.2	-9.08
	*Hsr 9*.*2*	C9	63.0	5.93	SF13994-Indel146	11.82	10.38
	*Hsr 9*.*3*	C9	111.0	3.44	BoE344-BoE975	8.67	-8.20

^**a**^ QTLs are named according to the trait (i.e., *Hsr*, head splitting resistance) followed by the chromosome number and position.

^**b**^ Proportion of the phenotypic variation explained by each QTL.

^**c**^ Additive effect: positive and negative values indicate that parental lines 96–100 and 79–156 respectively bear the head splitting resistance-enhancing allele.

## Discussion

HSR is an important agronomic trait, being associated with appearance, yield, mechanical harvestability, marketability, and storability. HSR evaluation method is the basis of further related research. Days to splitting after maturity or head splitting rate was most adopted as resistance evaluation criteria by relevant previous studies [5–6]. Although they are simple and intuitive, the characteristic of HSR can't be fully embody for different split layers and size. In our paper, the head splitting index which was scored based on split layers and size 15 days after maturity was adpoted to indicate the resistance to head splitting. It is a prerequisite basis for further studies about HSR in cabbage.

The relationship between splitting-tolerant characteristics and surface micro-configuration and cell tissue structure of leaf in cabbage has been reported [33, 51]. For splitting-resistant cultivars, the defensive cells were rounded cuticles of projected ridges, and epidermal cells were covered with dense and power while the anticlinal walls were sink in and form groove in splitting-susceptiblity cultivars, and the cuticles in periclinal walls were more smooth. Meanwhile, in splitting-resistant cultivars, mesophyll cells arranged tightly and small gap between cell. However, mesophyll cells had loose structure and big space between cells in splitting-susceptible ones. In our paper, we studied the correlations between Hsr and the other five head traits. There were negative and significant correlations between Hsr and Hvd, Htd, or Hw; however, there was no correlation between Hsr and Hm or Hsi.

### Genetic analysis

Qin et al. (1994) have reported that head splitting in cabbage under water-sufficient conditions is mainly due to genetic factors [4]. In the present study, ANOVA revealed significant differences in the head splitting index among DHs under the same cultivation conditions, indicating the existence of heritable variation, with significant interannual differences were also uncovered. These findings suggest that head splitting is mainly controlled by genetic features, with environmental conditions having a lesser influence. Similar results have been recorded for Chinese cabbage as well as watermelon, tomato, and various other fruit crops [52–53]. The development of varieties having HSR is thus an essential strategy for breeders.

Few relevant genetic analyses have been conducted, however, and previous genetic studies of HSR inheritance have focused only on HSR as a polygenic system and ignored the effect of individual genes [5–6]. To ensure the precision of genetic analyses for quantitative traits, the use of a segregating population with more than 100 individuals and several replications is required. In our study, we therefore performed a segregation analysis of the HSR trait using a DH population comprising more than 150 lines with three replications over 2 years. No cytoplasmic effect on HSR inheritance in cabbage was observed, and the head splitting index of the DH lines showed a continuous distribution. On the basis of genetic effects and gene heritability, HSR is inferred to be a complex quantitative trait regulated by the additive-epistatic effects of three major genes as well as polygenes. Additive effects predominated over all other types of genetic effects, and, as in previous studies, higher heritabilities of the trait were recorded with respect to major genes than polygenes [5–6]. HSR is primarily controlled by hereditable factors: more than 80.0% of the phenotypic variation among DH populations is controlled by three major genes and polygenes, with environmental factors having a minimal influence (< 10.0%).

### Linkage map construction

Genetic maps are of great significance in inheritance research, gene mapping, and function analysis. To date, a few genetic linkage maps for cabbage have been constructed. Most of these maps use different markers and have been based on F_2_ populations derived from inter-subspecies crosses [29, 54–55]. Recently, SSR, SNP, and InDel markers have greatly advanced with the development of next-generation sequencing technologies. InDel markers, compared to SSR and SNP, are attracting more and more attention in recent years because they can be obtained easily through bioinformatic methods based the massive re-sequencing data and also the electrophoresis bands are more reliable with single distinguishable bands of about100–200 bp, which reduces the genotyping errors greatly [27].

Populations of DH lines are ideal for the genetic analysis of quantitative traits, because DH populations are composed of genetically fixed DH lines. This situation allows the DH lines to be replicated between test sites and trialed over years, decreasing the standard error of QTL genotype means particularly true for *Brassica oleracea* where the diploid genome contains a high degree of gene replication and allowing a better estimate of trait heritability and increased power to detect QTLs [56]. The DH lines have only undergone one round of meiosis; therefore, the number of recombination events breaking disadvantageous linkages is reduced compared with an F_2_ or recombinant inbred line population. Nevertheless, the produced lines capture sufficient recombination events to be useful for calculating recombination fractions and thus marker linkage and map distances [57]. At the same time, the advantage of DH production over inbred lines produced by successive self-pollination is that only one round of recombination takes place, with advantageous gene combinations found in parental lines thus more likely to be preserved [58]. Although DH lines derived from specific crosses have been used to develop published linkage maps of *B*. *oleracea* [59–60], most of these lines were produced from crosses between different varieties, such as between broccoli and either cabbage or Chinese kale. Here, we report a new cabbage linkage map using SSR markers in a DH population of 157 lines derived from an intra-subspecies cross of cabbage. The generated framework map contains nine linkage groups covering 1065.9 cM of common marker regions [24, 43], enabling anchorage to the reference *B*. *oleracea* map. The successful generation of this map demonstrates that a comparative approach can boost marker coverage in regions related to a trait of interest [57]. Although the number of available polymorphic loci may be reduced using intra-crop crosses, such a strategy—as pointed out by Walley et al. (2012)—allows a direct relationship to be established between trait and crop type; the genetic variation captured in this fashion reduces the time required to incorporate important agronomic traits into elite breeding material [57]. DH lines that have good comprehensive characteristics can be directly used for breeding. Our newly constructed map and the generated DH lines are therefore not only important for research on related characteristics of cabbage, but will also contribute to the exchange of materials between laboratories and successive research in the future [24, 57].

However, dominance estimates cannot be estimated with a DH population compared to F_2_. F_2_ mapping populations are temporary, and are difficult to maintain for long-term and comparative studies. But F_2_ can be inbred to produce immortal recombinant populations that share the same benefits as DH populations, it takes more time and resources to accomplish this but the result is the same. In addition, segregation distortion estimates in Brassica DH populations tend to be much higher than what is observed in F_2_ derived populations [58]. The high percentage of skewed markers (58%) found in our study is comparable with the 65% skewed marker level observed by Voorrips et al. (1997) [61]. Such high percentages are common in DH populations, most likely because of selection during microspore culture and/or the ability to produce seed during regeneration and seed-bulking phases [24, 62–63], and while this doesn't necessarily impact the construction of the linkage map it can impact estimation of QTL effects.

### Strategic considerations and QTL research

With the advent of molecular markers, QTL mapping has become increasingly important in molecular breeding, and marker-assisted selection (MAS) and gene discovery are now widely used for the breeding of field crops and vegetables [11, 57, 63]. Although some QTLs have been identified in cabbage, research is still at a preliminary stage: QTL cloning has not yet been reported and functional analysis studies are rare [43]. To date, studies of QTLs in cabbage have focused mainly on disease resistance [28–30] and important morphological traits [31–32]. The use of different genetic models, algorithms, and mapping procedures can produce different mapping results, even for the same set of data [64]. Whole-genome scanning with multiple mapping procedures has thus been recommended for QTL mapping [65]. Using data obtained over multiple years increases environmental heterogeneity and also allows improved estimates of QTLs that may not reach the genome-wide significance threshold in just one environment [66–68]. For cabbage breeders, HSR is an important characteristic that affects both cabbage yield and quality. However, QTLs of HSR for cabbage has been only reported by Pang et al. using a genetic map constructed with an F_2_ population [33]. It was showed that a total of six quantitative trait loci (QTLs) conferring resistance to head splitting which were located in chromosome 2, 4, and 6. Two stable QTLs, *SPL-2-1* and *SPL-4-1*, on chromosomes 2 and 4, respectively, were detected in the experiments over 2 years. In our study, two mapping algorithms were used to analyze agronomic trait data collected during 2 years in a DH population. Our study results provide support for the above-mentioned assertions. Using the MQM algorithm, we identified six QTLs associated with HSR on chromosomes C4, C7, and C9 in cabbage over 2 years. Two robust QTLs with relatively large effects, *Hsr 4*.*2* and *Hsr 9*.*2*, were detected in both years, Using ICIM, seven QTLs distributed on chromosomes C3, C4, and C9, including the major QTLs *Hsr 4*.*2* and *Hsr 9*.2, were detected in both years. *Hsr 3*.*2*, which displayed a large effect, was aslo identified in both years while it was not detected by MQM. On chromosome C9, QTL *Hsr 9*.*1* with a larger effect is physically quite close to *Hsr 9*.*2*. Whether these QTLs are independent or instead both components of a larger QTL is an open question. The results of different mapping methods can potentially complement one another. For example, QTLs distributed on C7 and C3 were not identified by both methods; in addition, *Hsr 9*.*1* and *Hsr 9*.*2* could both be detected by ICIM and MQM, but *9*.*3* was identified only by ICIM. In brief, three major QTLs (*Hsr 3*.*2*, *Hsr 4*.*2*, *Hsr 9*.*2*) and some minor QTLs were detected over 2 years using the two mapping methods. Because this result is consistent with the results of classical genetic analysis, both segregation analysis and QTL mapping with molecular markers can be used as a mutual check and supplement [8]. Compared to the previous study [33], we identified some new loci associated with HSR for cabbage using different materials and QTL mapping methods. We also identified a stable QTL, *Hsr 4*.*2* on chromosomes C4, which is consistent with the findings of the previous study, indicating that chromosomes C4 is a major region controlling cabbage head splitting. Whether these two QTLs are the same loci needs to be further researched.

Although cabbage lines resistant to head splitting have traditionally been selected in the field during the mature stage, splitting severity depends on environmental factors and plant conditions. The use of DNA markers enables reliable selection of resistant plants, even at the seedling stage. DNA MAS has become popular for the breeding of crops, for which marker information is abundantly available. The QTLs identified in our study will be helpful for the identification of genes related to HSR and for MAS in cabbage breeding programs.

## Supporting Information

S1 Table Design of the field experiments. ^**a**^Parents, F_1_, and RF_1_ plants were distributed according to a randomized complete block design with three replicates. ^**b**^Doubled haploid (DH) lines were first divided randomly into 14 blocks in 2011 and 11 blocks in 2012; the blocks were then distributed according to a randomized complete block design with three replicates.
(DOC)

S2 Table Genetic models tested during joint segregation analysis of doubled haploid populations (adopted from Gai et al. 2003). ^**a**^B-1-X or E-1-X denote models without linkage, while B-2-X or E-2-X denote models with linkage. ^**b**^m, population mean; *d*, major gene additive effects for models A and D; *d*_*a*_, *d*_*b*_, and *d*_*c*_, additive effects of the first, second, and third major genes, respectively, for models B, E, F, and G; *i*, additive × additive effect of the two major genes for models B and E; *i*_*ab*_, *i*_*ac*_, *i*_*bc*_, and *i*_*abc*_, interaction effect of the first and second major genes, the first and third major genes, the second and third major genes, and the three major genes, respectively, for models F and G; i*, includes additive and additive × additive effects.
(DOC)

S3 Table Akaike information criterion (AIC) values estimated for different genetic models. ^**a**^ Underlined models were selected as candidate models because of their smaller AIC values.
(DOCX)

S4 Table Tests for goodness of fit of alternative models. ^**a**^ *U*_1_^2^, *U*_2_^2^, and *U*_3_^2^, *χ* ^2^ statistics; n*W* ^2^, Smirnov’s statistic; *D*_*n*_, Kolmogorov’s statistic. Values in parentheses after *U*_1_^2^, *U*_2_^2^, *U*_3_^2^, and *D*_*n*_ values are probabilities; values of n*W* ^2^ are 0.461 and 0.743 at *P* < 0.05 and *P* < 0.01 significance levels, respectively. ^**b**^Underlined values are significant.
(DOC)
