# Supplementary material for: QTL Analysis of Head Splitting Resistance in Cabbage (Brassica oleracea L. var. capitata) Using SSR and InDel Makers Based on Whole-Genome Re-Sequencing
Source: PLoS One. 2015 Sep 25;10(9):e0138073. doi: 10.1371/journal.pone.0138073 (PMC4583274; doi:10.1371/journal.pone.0138073)
Supplement: S1 Table — aParents, F1, and RF1 plants were distributed according to a randomized complete block design with three replicates. bDoubled haploid (DH) lines were first divided randomly into 14 blocks in 2011 and 11 blocks in 2012; the blocks were then distributed according to a randomized complete block design with three replicates. (DOC) [file pone.0138073.s001.doc]

**Table S1 Design of the field experiments**

| Year | The order of parents, F_1_, RF_1_ and the block number in each repeat of DHs | | | | | | | | | | | | | | | | | | |
| --- | --- | --- | --- | --- | --- | --- | --- | --- | --- | --- | --- | --- | --- | --- | --- | --- | --- | --- | --- |
| 2011 | I | P_1_**^a^** | P_2_ | F_1_ | RF_1_ | 1**^b^** | 6 | 3 | 9 | 2 | 14 | 10 | 11 | 4 | 12 | 8 | 5 | 13 | 7 |
|  | II | P_2_ | P_1_ | RF_1_ | F_1_ | 2 | 9 | 14 | 7 | 1 | 10 | 8 | 4 | 6 | 11 | 5 | 13 | 3 | 12 |
|  | III | P_1_ | F_1_ | RF_1_ | P_2_ | 14 | 5 | 12 | 4 | 8 | 1 | 10 | 6 | 9 | 13 | 3 | 7 | 2 | 11 |
| 2012 | I | P_1_ | P_2_ | F_1_ | RF_1_ | 1 | 5 | 6 | 11 | 9 | 7 | 2 | 3 | 10 | 4 | 8 |  |  |  |
|  | II | F_1_ | P_1_ | RF_1_ | P_2_ | 6 | 9 | 7 | 2 | 10 | 8 | 4 | 1 | 5 | 11 | 3 |  |  |  |
|  | III | P_1_ | RF_1_ | F_1_ | P_2_ | 3 | 5 | 11 | 4 | 8 | 10 | 6 | 9 | 1 | 7 | 2 |  |  |  |

**^a^** Parents, F_1_, and RF_1_ plants were distributed according to a randomized complete block design with three replicates.

**^b^** Doubled haploid (DH) lines were first divided randomly into 14 blocks in 2011 and 11 blocks in 2012; the blocks were then distributed according to a randomized complete block design with three replicates.
