# Supplementary material for: QTL Analysis of Head Splitting Resistance in Cabbage (Brassica oleracea L. var. capitata) Using SSR and InDel Makers Based on Whole-Genome Re-Sequencing
Source: PLoS One. 2015 Sep 25;10(9):e0138073. doi: 10.1371/journal.pone.0138073 (PMC4583274; doi:10.1371/journal.pone.0138073)
Supplement: S2 Table — aB-1-X or E-1-X denote models without linkage, while B-2-X or E-2-X denote models with linkage. bm, population mean; d, major gene additive effects for models A and D; d a, d b, and d c, additive effects of the first, second, and third major genes, respectively, for models B, E, F, and G; i, additive × additive effect of the two major genes for models B and E; i ab, i ac, i bc, and i abc, interaction effect of the first and second major genes, the first and third major genes, the second and third major genes, and the three major genes, respectively, for models F and G; i*, includes additive and additive × additive effects. (DOC) [file pone.0138073.s002.doc]

**Table S2 Genetic models tested during joint segregation analysis of doubled haploid populations (adopted from Gai et al. 2003)**

| Pairs of  major gene  major gene | Model | Composition  Distribution  proportion | Composition  distribution  average | Model type **^a^** | | Parameters estimated**^b^** | |
| --- | --- | --- | --- | --- | --- | --- | --- |
|  |  |  |  | Only major gene | Major gene and polygene | Major gene | Polygene |
| 0 | - | - | *µ*_1_=*m* | A-0 | C | - | [*d*] |
| 1 | Additive | 1 | *µ*_1_=*m*+*d*  *µ*_2_=*m*-*d* | A-1 | D1 | *m*, *d* | [*d*] |
| 2 | Additive-epistasis | Equal | *µ_1_=m*+*d_a_*+*d_b_*+*i*  *µ_2_*=*m*+*d_a_*-*d_b_*-*i*  *µ_3_=m-d_a_+d_b_-i*  *µ_4_*=*m*-*d_a_*-*d_b_*+*i* | B-1-1 | E-1-1 | *m*, *d_a_*, *d_b_*, *i* | [*d*] |
|  | Additive | Equal | *µ_1_=m*+*d_a_*+*d_b_* *µ_2_*=*m*+*d_a_*-*d_b_*  *µ_3_=m-d_a_+d_b_ µ_4_*=*m*-*d_a_*-*d_b_* | B-1-2 | E-1-2 | *m*, *d_a_*, *d_b_* | [*d*] |
|  | Equal additive | 1:2:1 | *µ*_1_=*m+*2*d µ*_2_=*m*  *µ*_3_=*m*-2*d* | B-1-3 | E-1-3 | *m,* *d*=*d_a_*=*d_b_* | [*d*] |
|  | Dominant epistasis | 1:2:1 | *µ*_1_=*m+d_a_*  *µ*_2_=*m*- *d_a_*+*d_b_*  *µ*_3_= *m*- *d_a_*-*d_b_* | B-1-4 | E-1-4 | *m*, *d_a_*, *d_b_* | [*d*] |
|  | Recessive epistasis | 1:1:2 | *µ*_1_=*m*+*d_a_*+*d_b_ µ*_2_=*m*+*d_a_*- *d_b_*  *µ*_3_= *m*-*d_a_* | B-1-5 | E-1-5 | *m*, *d_a_*, *d_b_* | [*d*] |
|  | cumulative | 1:2:1 | *µ*_1_=*m+*2*d*+*i*  *µ*_2_=*m*-*i*  *µ*_3_=*m*-2*d*+*i* | B-1-6 | E-1-6 | *m*, *d*=*d_a_*=*d_b_, i* | [*d*] |
|  | Complementary | 1:3 | *µ*_1_=*m*+*i*^*^  *µ*_2_=*m*-*i^*^* | B-1-7 | E-1-7 | *m*, *i^*^* | [*d*] |
|  | Duplicate | 3:1 | *µ*_1_=*m*+*i*^*^  *µ*_2_=*m*-*i^*^* | B-1-8 | E-1-8 | *m*, *i^*^* | [*d*] |
|  | Inhibitory | 3:1 | *µ*_1_=*m*-*i*^*^  *µ*_2_=*m*+*i^*^* | B-1-9 | E-1-9 | *m*, *i^*^* | [*d*] |
| 3 | Additive-epistasis | Equal | *µ*_1_~*µ*_8_ | F-1 | G-1 | *m*, *d_a_*, *d_b_*, *d_c_*, *i_ab_*,  *i_ac_*, *i_bc_*, *i_abc_* | [*d*] |
|  | Additive | Equal | *µ*_1_~*µ*_8_ | F-2 | G-2 | *m*, *d_a_*, *d_b_*, *d_c_* | [*d*] |
|  | Equai additive(1) | 1:3: 3:1 | *µ*_1_~*µ*_4_ | F-3 | G-3 | *m*, *d*=*d_a_*=*d_b_*=*d_c_* | [*d*] |
|  | Equai additive(2) | 1:1 :2 :2 :1:1 | *µ*_1_~*µ*_6_ | F-4 | G-4 | *m*,*d*_1_=*d_a_*=*d_b_*,  *d*_2_=*d_c_* | [*d*] |

**^a^** B-1-X or E-1-X denote models without linkage, while B-2-X or E-2-X denote models with linkage.

**^b^** m, population mean; *d*, major gene additive effects for models A and D; *d_a_*, *d_b_*, and *d_c_*, additive effects of the first, second, and third major genes, respectively, for models B, E, F, and G; *i*, additive × additive effect of the two major genes for models B and E; *i_ab_*, *i_ac_*, *i_bc_*, and *i_abc_*, interaction effect of the first and second major genes, the first and third major genes, the second and third major genes, and the three major genes, respectively, for models F and G; *i^*^*, includes additive and additive × additive effects.
