# Supplementary material for: QTL Analysis of Head Splitting Resistance in Cabbage (Brassica oleracea L. var. capitata) Using SSR and InDel Makers Based on Whole-Genome Re-Sequencing
Source: PLoS One. 2015 Sep 25;10(9):e0138073. doi: 10.1371/journal.pone.0138073 (PMC4583274; doi:10.1371/journal.pone.0138073)
Supplement: S3 Table — a Underlined models were selected as candidate models because of their smaller AIC values. (DOCX) [file pone.0138073.s003.docx]

**Table 3** Akaike information criterion (AIC) values estimated for different genetic models

| Model | AIC | | Model | AIC | |
| --- | --- | --- | --- | --- | --- |
|  | 2011 | 2012 |  | 2011 | 2012 |
| A-0 | 1576.176 | 1829.380 | E-2-0 | 1442.933 | 1724.153 |
| A-1 | 1477.031 | 1733.070 | E-2-1 | 1442.181 | 1722.841 |
| B-1-1 | 1448.086 | 1736.838 | E-2-2 | 1467.829 | 1722.419 |
| B-1-2 | 1439.054 | 1678.730**^a^** | E-2-3 | 1446.232 | 1692.444 |
| B-1-3 | 1464.138 | 1695.314 | E-2-4 | 1440.953 | 1721.049 |
| C-0 | 1533.019 | 1778.552 | E-2-5 | 1440.953 | 1721.049 |
| C-1 | 1541.363 | 1788.985 | E-2-6 | 1465.257 | 1720.842 |
| D-0 | 1464.339 | 1719.479 | E-2-7 | 1465.042 | 1719.195 |
| D-1 | 1463.836 | 1718.423 | E-2-8 | 1465.042 | 1719.196 |
| E-1-0 | 1440.924 | 1723.253 | E-2-9 | 1534.623 | 1779.918 |
| E-1-1 | 1440.172 | 1721.397 | F-1 | 1438.600 | 1683.141 |
| E-1-2 | 1465.832 | 1720.422 | F-2 | 1426.862 | 1708.692 |
| E-1-3 | 1453.709 | 1689.240 | F-3 | 1444.692 | 1691.289 |
| E-1-4 | 1438.942 | 1719.573 | F-4 | 1558.399 | 1713.401 |
| E-1-5 | 1438.942 | 1719.573 | G-0 | 1434.154 | 1677.556 |
| E-1-6 | 1463.393 | 1719.397 | G-1 | 1433.911 | 1678.578 |
| E-1-7 | 1463.114 | 1717.723 | G-2 | 1465.797 | 1720.432 |
| E-1-8 | 1463.114 | 1717.723 | G-3 | 1461.501 | 1779.675 |
| E-1-9 | 1532.740 | 1778.148 | G-4 | 1455.627 | 1691.234 |

**^a^** Underlined models were selected as candidate models because of their smaller AIC values.
