# Supplementary material for: QTL Analysis of Head Splitting Resistance in Cabbage (Brassica oleracea L. var. capitata) Using SSR and InDel Makers Based on Whole-Genome Re-Sequencing
Source: PLoS One. 2015 Sep 25;10(9):e0138073. doi: 10.1371/journal.pone.0138073 (PMC4583274; doi:10.1371/journal.pone.0138073)
Supplement: S4 Table — a U 1 2, U 2 2, and U 3 2, χ 2 statistics; nW 2, Smirnov’s statistic; D n, Kolmogorov’s statistic. Values in parentheses after U 1 2, U 2 2, U 3 2, and D n values are probabilities; values of nW 2 are 0.461 and 0.743 at P < 0.05 and P < 0.01 significance levels, respectively. bUnderlined values are significant. (DOC) [file pone.0138073.s004.doc]

Table S3 Tests for goodness of fit of alternative models

| Year | Model | Generation | *U*_1_^2^**^a^** | *U*_2_^2^ | *U*_3_^2^ | _n_*W*^2^ | *D*_n_ | No. of significant parameter(s) |
| --- | --- | --- | --- | --- | --- | --- | --- | --- |
| 2011 | F-2 | P1 | 1.65(0.20) | 1.82(0.18) | 0.18(0.68) | 0.23(>0.05) | 0.58 (>0.05) | 5 |
|  |  | P2 | 0.35(0.55) | 0.04(0.84) | 2.20(0.14) | 0.15(>0.05) | 0.47(>0.05) |
|  |  | DH | 16.30(0.00)^b^ | 10.47(0.00) | 7.28(0.01) | 2.16(<0.01) | 0.21(<0.05) |
|  | G-0 | P1 | 0.09(0.77) | 0.34(0.56) | 1.42(0.23) | 0.10(>0.05) | 0.41(>0.05) | 0 |
|  |  | P2 | 0.00(0.97) | 0.02(0.90) | 0.42(0.52) | 0.04(>0.05) | 0.24(>0.05) |
|  |  | DH | 0.09(0.76) | 0.02(0.90) | 0.42(0.52) | 0.11(>0.05) | 0.09(>0.05) |
|  | G-1 | P1 | 0.00(0.96) | 0.07(0.79) | 3.27(0.07) | 0.08(>0.05) | 0.35(>0.05) | 4 |
|  |  | P2 | 0.10(0.76) | 0.22 (0.64) | 0.48(0.49) | 0.05(>0.05) | 0.30 (>0.05) |
|  |  | DH | 4.82(0.03) | 6.64 (0.01) | 3.27(0.07) | 0.55(<0.05) | 0.11 (<0.05) |
|  | F-1 | P1 | 0.00(0.96) | 0.17 (0.68) | 2.15 (0.14) | 0.10(>0.05) | 0.38 (>0.05) | 4 |
|  |  | P2 | 0.16(0.69) | 0.65 (0.42) | 2.92(0.09) | 0.16(>0.05) | 0.49(>0.05) |
|  |  | DH | 4.94(0.03) | 6.68 (0.01) | 3.00 (0.08) | 0.55(<0.05) | 0.11 (<0.05) |
| 2012 | G-0 | P1 | 0.09(0.77) | 0.34 (0.56) | 1.42 (0.23) | 0.10(>0.05) | 0.41 (>0.05) | 1 |
|  |  | P2 | 0.00(0.97) | 0.02 (0.90) | 0.42 (0.52) | 0.04(>0.05) | 0.24 (>0.05) |
|  |  | DH | 2.88(0.09) | 3.31 (0.07) | 0.51(0.47) | 0.43(>0.05) | 0.10 (<0.05) |
|  | G-1 | P1 | 0.12(0.73) | 0.07 (0.79) | 0.07(0.78) | 0.07(>0.05) | 0.31 (>0.05) | 1 |
|  |  | P2 | 0.61(0.43) | 0.84 (0.36) | 0.42(0.52) | 0.10(>0.05) | 0.41(>0.05) |
|  |  | DH | 3.42(0.06) | 3.73 (0.05) | 0.32(0.57) | 0.45(>0.05) | 0.10 (<0.05) |
|  | B-1-2 | P1 | 0.51(0.47) | 1.04 (0.31) | 1.72(0.19) | 0.16(>0.05) | 0.52(>0.05) | 5 |
|  |  | P2 | 0.14(0.70) | 0.65(0.42) | 3.06(0.08) | 0.17 (>0.05) | 0.50(>0.05) |
|  |  | DH | 35.75(0.00) | 24.59(0.00) | 11.03(0.00) | 3.98(<0.01) | 0.27(<0.05) |
|  | F-1 | P1 | 0.76(0.38) | 1.29(0.26) | 1.36(0.24) | 0.18(>0.05) | 0.55(>0.05) | 1 |
|  |  | P2 | 0.13(0.72) | 0.62(0.43) | 3.09(0.08) | 0.17(>0.05) | 0.49 (>0.05) |
|  |  | DH | 3.01(0.08) | 3.51(0.06) | 0.60(0.44) | 0.45(>0.05) | 0.11(<0.05) |

**^a^** *U*_1_^2^, *U*_2_^2^, and *U*_3_^2^, *χ*^2^ statistics; n*W*^2^, Smirnov’s statistic; *D_n_*, Kolmogorov’s statistic. Values in parentheses after *U*_1_^2^, *U*_2_^2^, *U*_3_^2^, and *D_n_* values are probabilities; values of n*W*^2^ are 0.461 and 0.743 at *P* < 0.05 and *P* < 0.01 significance levels, respectively.

**^b^** Underlined values are significant.
